# Development and validation of a machine learning model for predicting hypersplenism in Wilson disease patients

**DOI:** 10.3389/fmed.2026.1768024

**Published:** 2026-03-18

**Authors:** Qiaoyu Xuan, Xiuquan Shi, Lei Jin, Daiping Hua, Lanting Sun, Wenming Yang, Han Wang

**Affiliations:** 1 Department of Neurology, The First Affiliated Hospital of Anhui University of Chinese Medicine, Hefei, China; 2 Department of General Surgery, Yingshang County People's Hospital, Yingshang, China; 3 Key Laboratory of Xin'An Medicine, Ministry of Education, Anhui University of Chinese Medicine, Hefei, China

**Keywords:** hypersplenism, LASSO regression, machine learning, predictive model, SHAP analysis, Wilson disease

## Abstract

**Objective:**

Wilson disease (WD) is a rare autosomal recessive copper metabolism disorder, with hypersplenism as a severe, common complication secondary to disease-related cirrhosis. Currently, there is a lack of precise early prediction tools for this complication. This study aimed to construct a hypersplenism prediction model for WD patients by integrating multidimensional clinical indicators and machine learning, providing references for early identification of high-risk individuals and personalized interventions.

**Methods:**

A total of 524 WD patients were enrolled at the First Affiliated Hospital of Anhui University of Chinese Medicine from December 2019 to February 2025, including 244 with hypersplenism (HG) and 280 without (non-HG). After Key variables were selected through LASSO regression feature selection. Variate multicollinearity within the model was assessed using variance inflation factors (VIF). The predictive model was visualized using a nomogram. Five machine learning models were built with 10-fold cross-validation for parameter optimization. Finally, the model performance was evaluated, and the feature contributions were explained using the SHapley Additive exPlanations (SHAP) method.

**Results:**

Compared with the non-HG group, the HG group had significantly lower WBC, PLT, and ceruloplasmin (CER), and higher A/G, PIIINP, CIV, hyaluronic acid (HA), laminin (LN), and 24-h urinary copper (CUU) (all *p* < 0.05). Multivariate logistic regression showed A/G, CIV, and PIIINP were independent risk factors, while WBC and PLT were independent protective factors. The SVM model performed best: training set AUC = 0.867 (95% CI: 0.830–0.904), accuracy = 0.807, specificity = 0.856, precision = 0.812, F1 score = 0.771; test set AUC = 0.771 (95% CI: 0.699–0.844) with AUC decay <10%. It also had excellent calibration (training set Brier score = 0.146, test set = 0.206) and clinical utility via DCA. SHAP analysis identified PIIINP as the core predictive feature, followed by WBC, PLT, and A/G, with CIV having relatively weaker influence.

**Conclusion:**

The SVM-based predictive model exhibits superior discriminatory power, calibration accuracy, and clinical utility for hypersplenism in WD patients. The five key features (WBC, PLT, A/G, CIV, PIIINP) with PIIINP as the core provide an objective quantitative basis for risk stratification, facilitating early identification and precise intervention of high-risk patients and improving WD prognosis.

## 1 Introduction

Wilson disease (WD) is an autosomal recessive disorder of copper metabolism caused by mutations in the ATP7B gene (1). Dysfunction of the transmembrane copper transport ATPase encoded by this gene directly impairs the normal excretion of copper ions into bile or their binding to ceruloplasmin. This leads to progressive accumulation of copper in vital organs such as the liver and brain, triggering multisystem damage through mechanisms including oxidative stress and cytotoxicity (2). As the primary target organ where disease originates, the pathological progression of the liver follows a distinct sequential pattern: beginning with early-stage hepatocyte inflammation and steatosis, gradually advancing to hepatic fibrosis, and ultimately progressing to cirrhosis (3). Portal hypertension secondary to liver cirrhosis readily induces hypersplenism, a severe complication. Together with liver failure and significant neurological impairment, these three core factors constitute the primary determinants of poor prognosis in WD patients, directly impacting long-term survival (4). WD exhibits significant clinical heterogeneity, with manifestations ranging from asymptomatic hepatic involvement to severe neuropsychiatric symptoms, further complicating early risk stratification and individualized management (5).

Hypersplenism, as a characteristic complication of portal hypertension associated with WD-related cirrhosis, primarily manifests as splenomegaly accompanied by hematological abnormalities such as thrombocytopenia and leukopenia (6). This has dual implications for clinical management: on one hand, cytopenia substantially increases the risk of spontaneous bleeding and opportunistic infections in patients, representing the primary cause of acute adverse events in WD patients (7, 8). On the other hand, toxic alterations in the hematopoietic system limit the use of copper chelators—drugs central to the etiological treatment of WD. Inadequate dosing or delayed administration can lead to persistent copper accumulation, creating a vicious cycle of “complications—treatment limitations—disease progression.” Currently, clinical prognostic assessment tools such as the Child-Pugh score and the Model for End-Stage Liver Disease (MELD) are widely used for evaluating liver function in chronic liver diseases (9). However, these tools are not specifically tailored to the pathophysiological characteristics of WD and demonstrate limited predictive accuracy for hypersplenism-related adverse outcomes. The lack of a targeted prognostic tool hinders early identification of high-risk patients and optimal individualized management.

The rise of machine learning (ML) technology offers a new pathway to address this clinical challenge. Its core advantage lies in capturing complex nonlinear relationships among multidimensional clinical indicators (10), which is particularly crucial for prognostic modeling in highly heterogeneous diseases like WD. ML-based models have achieved promising performance in predicting outcomes for various chronic liver diseases, including cirrhosis progression and complication risks (11). However, to date, no dedicated ML prediction model has been developed specifically for the risk of splenomegaly in WD patients. Existing studies have primarily focused on single biomarkers or imaging indicators, presenting limitations such as low accessibility and narrow predictive dimensions. Based on this, this study integrates routine clinical and laboratory indicators from WD patients to construct multiple ML prediction models. Simultaneously, it introduces the SHapley Additive exPlanations (SHAP) framework to quantify the contribution of each feature, thereby addressing the “black box” challenge of ML models. The study aims to develop a precise, interpretable, and easily transferable splenomegaly risk prediction tool, providing scientific evidence for early intervention and personalized management of WD patients, ultimately improving patient prognosis.

## 2 Materials and methods

### 2.1 Study design and population

A retrospective case–control study was conducted, including 524 patients diagnosed with WD at the First Affiliated Hospital of Anhui University of Chinese Medicine between December 2019 anFebruary 2025. All patients met the criteria outlined in the European Association for the Study of the Liver (EASL) Clinical Practice Guidelines for WD (4). Hypersplenism is defined as: a platelet count <75 × 10^9^/L and/or a white blood cell count <3.5 × 10^9^/L (12). Based on the above definitions, patients were categorized into the hypersplenism group (HG) and the non-hypersplenism group (non-HG). Additionally, the following predictive factor information was collected via the electronic medical record system: gender, age, clinical phenotype, disease duration, complete blood count, liver function, liver fibrosis, coagulation function, ceruloplasmin and 24-h urinary copper (CUU) levels. Clinical phenotypes are classified as liver, brain, or mixed based on the predominant symptom at initial presentation, following the EASL guidelines. Disease duration is defined as the interval from the date of diagnosis to the date of enrollment.

Exclusion criteria included: (1) Coexisting other liver diseases (e.g., viral hepatitis, alcoholic liver disease, non-alcoholic fatty liver disease); (2) Comorbid malignancies (e.g., hepatocellular carcinoma, extrahepatic tumors); (3) Post-splenectomy or severe lack of clinical data (> 30% key variables missing); (4) Pregnancy or lactation; (5) Documented primary bone marrow disorders (e.g., aplastic anemia, myelodysplastic syndrome, leukemia) or ongoing myelosuppressive therapy (e.g., chemotherapy, immunosuppressants) that could independently cause cytopenia.

This study was conducted following the 1964 Declaration of Helsinki and approved by the Ethics Committee of the First Affiliated Hospital of Anhui University of Traditional Chinese Medicine (Approval No.: 2025AH-143-01). All subjects received written informed consent.

### 2.2 Data processing

This dataset was divided using stratified random sampling, with 70% of the data allocated to the training set and 30% to the independent test set. This approach prevents sampling bias caused by uneven distribution of the outcome variable. Missing data handling: (1) Variables with <10% missing values: Imputed by median (continuous variables) or mode (categorical variables); (2) Variables with 10–30% missing values: Imputed via multivariate imputation by chained equations (MICE) with 5 imputed datasets. Outlier handling was conducted after missing value imputation. The 1st and 99th percentiles of the training set were used as truncation thresholds to avoid data leakage.

**Figure 1:**
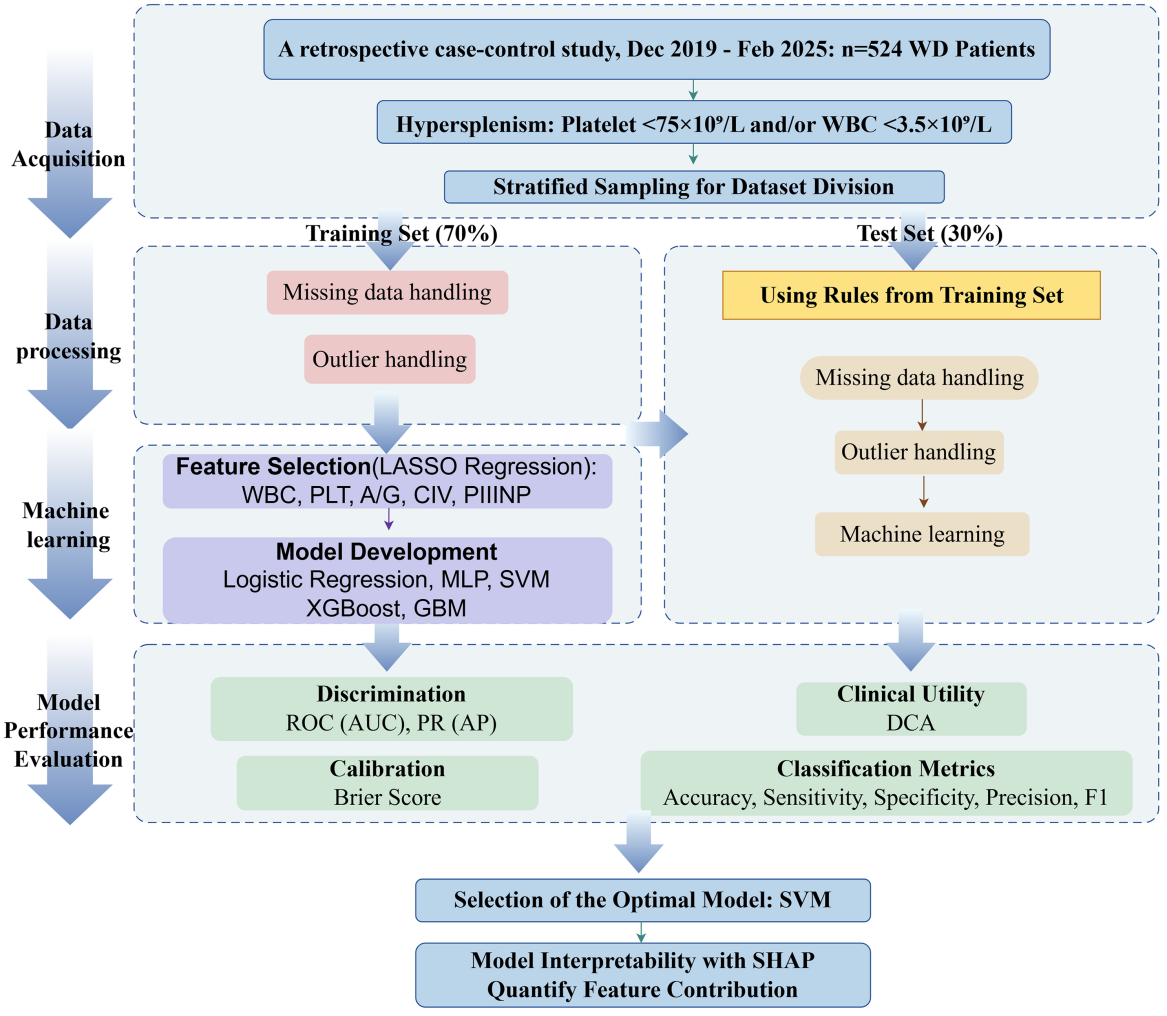
Flowchart for constructing and validating a prediction model for splenomegaly in patients with Wilson disease.

The Least Absolute Shrinkage and Selection Operator (LASSO) algorithm was applied to the training set to select meaningful feature variables for inclusion in the model. Subsequently, multivariate logistic regression, multilayer perceptron (MLP), support vector machine (SVM), extreme gradient boosting (XGBoost), and gradient boosting machine (GBM) to predict splenomegaly in WD patients. Ten-fold cross-validation was employed to determine optimal parameters for each model, followed by independent validation on the test set. Receiver operating characteristic (ROC) curves were plotted to assess model discrimination. The SHAP algorithm was applied to the best-performing model for interpretability analysis, enhancing model transparency. The technical workflow is illustrated in Figure 1.

### 2.3 Statistical analysis

Since this study involves a binary classification problem, the Events Per Variable (EPV) method is suitable for estimating sample size. The final number of features included in the multivariate regression model was 5, with 244 splenomegaly events. The EPV was approximately 48.8, significantly exceeding the recommended threshold of EPV > = 10–15. This indicates the model possesses good stability and a low risk of overfitting (13).

Continuous variables that follow a normal distribution are expressed as mean ± standard deviation; non-compliant variables are represented by median and interquartile range. Categorical variables are presented as frequency and percentage. For normally distributed continuous variables, t-tests were used for intergroup comparisons; Mann–Whitney U tests were applied for non-normally distributed continuous variables. Categorical variables were analyzed using chi-square tests or Fisher’s exact tests. All statistical analyses adhered to the principle of two-tailed testing with a significance level set at *α* = 0.05. Differences were considered statistically significant when *p* < 0.05. All data analysis for this study was performed using the R programming environment (version 4.5.2). Calculate the area under the receiver operating characteristic curve (AUC) as a model evaluation metric. Model evaluation metrics included area under the receiver operating characteristic curve (AUC), accuracy, sensitivity, specificity, precision, and F1 score. Ninety-five percent confidence intervals (95% CI) for all metrics were calculated via Bootstrap resampling (R = 50) to ensure reliability. Results.

## 3 Results

### 3.1 WD patient baseline characteristics

There were no significant differences in age and gender distribution between the two patient groups (*p* > 0.05). Compared with the non-hypersplenism (HG) group, patients in the HG group (hypersplenism group) exhibited significantly reduced levels of white blood cell (WBC), platelets (PLT), and ceruloplasmin (CER) (*p* < 0.05). Conversely, the HG group exhibited significantly elevated levels of alpha/gamma ratio (A/G), type III procollagen peptide (PIIINP), type IV collagen (CIV), hyaluronic acid (HA), laminin (LN), and urinary copper (CUU) (*p* < 0.05). Detailed baseline characteristics are shown in Table 1.

**Table 1:** Baseline characteristics of the study population stratified by clinical outcome.

Variable	Overview (*n* = 524)	non-HG (*n* = 280)	HG (*n* = 244)	*p*
Male (%)	330 (62.977)	171 (61.071)	159 (65.164)	0.380
Female (%)	194 (37.023)	109 (38.929)	85 (34.836)	
Age (year)	26.000 [20.000, 33.000]	27.000 [20.000, 33.000]	25.000 [19.000, 32.000]	0.454
Liver type (%)	308 (58.779)	164 (58.571)	144 (59.016)	0.963
Brain type (%)	145 (27.672)	77 (27.500)	68 (27.869)	
Mixed type (%)	71 (13.550)	39 (13.929)	32 (13.115)	
Disease Duration (months)	35.850 [23.00, 50.200]	35.150 [21.175, 48.725]	36.400 [24.300, 51.800]	0.263
WBC (10∧9/L)	4.625 [3.605, 5.682]	5.170 [4.188, 6.403]	4.105 [3.202, 4.860]	<0.001
RBC (10∧12/L)	4.500 [4.190, 4.950]	4.495 [4.250, 4.902]	4.505 [4.165, 4.962]	0.693
Hb (g/L)	133.000 [121.000, 144.000]	132.000 [122.00, 143.000]	134.000 [120.000, 144.000]	0.824
PLT (10∧9/L)	143.500 [101.000, 200.250]	175.500 [127.000, 242.500]	119.500 [88.750, 157.000]	<0.001
ALB (g/L)	39.600 [37.300, 41.725]	39.700 [37.400, 41.700]	39.550 [37.100, 41.925]	0.767
ALT (U/L)	25.000 [15.075, 43.000]	26.000 [15.000, 51.250]	23.500 [15.325, 35.750]	0.065
AST (U/L)	24.300 [19.675, 34.000]	25.000 [19.000, 36.000]	24.000 [20.000, 31.000]	0.415
GGT (U/L)	28.000 [18.000, 46.000]	27.000 [18.000, 46.000]	29.000 [18.000, 46.000]	0.537
TBA (μmol/L)	6.700 [4.200, 10.800]	6.400 [3.900, 10.350]	7.400 [4.500, 11.025]	0.054
TBIL (μmol/L)	13.690 [10.600, 18.800]	13.300 [10.275, 17.800]	14.000 [11.175, 19.400]	0.105
DBIL (μmol/L)	3.200 [2.300, 4.300]	3.100 [2.268, 4.400]	3.300 [2.500, 4.200]	0.657
IBIL (μmol/L)	10.450 [7.900, 14.325]	10.200 [7.537, 13.825]	10.900 [8.395, 15.000]	0.056
A/G	1.650 [1.450, 1.870]	1.600 [1.420, 1.763]	1.735 [1.490, 1.940]	<0.001
ALP (U/L)	102.000 [80.000, 140.000]	105.000 [80.000, 157.000]	99.500 [80.750, 122.000]	0.072
BUN (mmol/L)	4.925 [4.038, 5.860]	4.895 [3.975, 5.740]	4.935 [4.108, 5.875]	0.259
PT (s)	11.400 [10.800, 12.000]	11.400 [10.800, 11.925]	11.400 [10.900, 12.100]	0.084
INR	1.020 [0.960, 1.080]	1.020 [0.970, 1.080]	1.020 [0.960, 1.080]	0.579
FIB (g/L)	2.050 [1.790, 2.380]	2.090 [1.800, 2.400]	2.030 [1.775, 2.345]	0.203
CIV (ng/mL)	60.150 [42.195, 83.685]	50.310 [33.995, 69.830]	72.055 [51.902, 92.942]	<0.001
HA (ng/mL)	93.035 [57.250, 160.855]	69.505 [48.288, 120.380]	126.685 [79.158, 187.225]	<0.001
LN (ng/mL)	99.770 [80.130, 126.195]	96.760 [74.230, 125.147]	104.265 [84.228, 126.195]	0.031
PIIINP (ng/mL)	13.165 [8.775, 21.730]	11.790 [7.262, 17.205]	17.515 [10.532, 25.407]	<0.001
CER (g/L)	0.026 [0.023, 0.044]	0.029 [0.026, 0.049]	0.026 [0.014, 0.040]	<0.001
CUU (ug/24 h)	790.960 [418.720, 1286.815]	656.715 [393.618, 1171.797]	931.075 [495.680, 1357.793]	0.002

n, number; HG, hypersplenism group; non-HG, non-hypersplenism group; WBC, white blood cell; RBC, red blood cell; Hb, hemoglobin; PLT, platelet; ALB, albumin; ALT, alanine aminotransferase; AST, aspartate aminotransferase; GGT, gamma-glutamyl transferase; TBA, total bile acid; TBIL, total bilirubin; DBIL, direct bilirubin; IBIL, indirect bilirubin; A/G, albumin/globulin ratio; ALP, alkaline phosphatase; BUN, blood urea nitrogen; PT, prothrombin time; INR, international normalized ratio; FIB, fibrinogen; CIV, type IV collagen; HA, hyaluronic acid; LN, laminin; PIIINP, type III procollagen peptide; CER, ceruloplasmin; CUU, 24-h urinary copper.

### 3.2 Prediction model performance comparison

**Figure 2:**
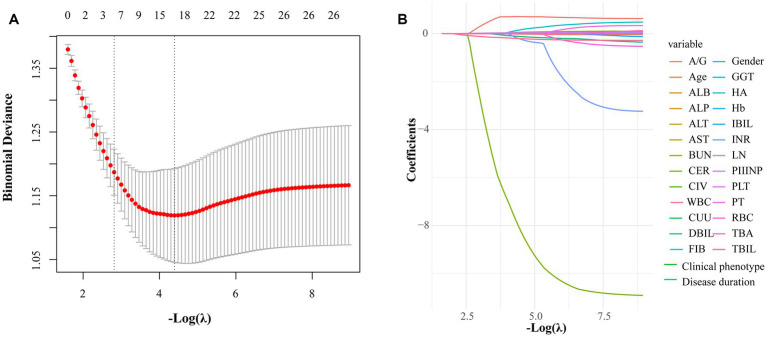
LASSO regression-based feature selection for hypersplenism in Wilson disease patients. **(A)** Cross-validation curve of LASSO regression. **(B)** Coefficient trajectory of binomial LASSO regression.

LASSO regression was employed to screen candidate predictors for splenomegaly in WD patients. The penalty parameter *λ* was optimized via 10-fold cross-validation (10-CV). Cross-validation results indicated that the logarithm of *λ*-min (corresponding to the minimum cross-validation error) was logλ = −4.393. To further mitigate overfitting risk and enhance model parsimony, *λ*-1SE was ultimately selected as the optimal penalty parameter. As −log(λ) increased, redundant variables were compressed to zero by the LASSO penalty term at λ-1SE, while non-redundant variables with stable predictive value retained non-zero coefficients (Figure 2).

Subsequently, the potential factors screened by the LASSO regression were incorporated into a multivariate binary logistic regression model. The WBC (*p* = 0.006), PLT (*p* < 0.001), A/G (*p* = 0.002), CIV (*p* = 0.019), and PIIINP (*p* < 0.001) were significantly associated with splenomegaly in WD patients (Table 2). Based on Odds Ratio (OR) and 95% confidence interval (CI) analysis: A/G, CIV, and PIIINP were independent risk factors (OR > 1 and 95% CI did not include 1); WBC and PLT were independent protective factors (OR < 1 and 95% CI did not include 1).

**Table 2:** Multivariate logistic regression analysis of factors associated with splenomegaly in Wilson disease.

Variable	Multivariate analysis (95%CI)	*p*
WBC	0.786 (0.656–0.926)	0.006
PLT	0.991 (0.986–0.995)	< 0.001
A/G	3.377 (1.607–7.344)	0.002
CIV	1.009 (1.002–1.017)	0.019
PIIINP	1.042 (1.021–1.065)	< 0.001

### 3.3 Predictive model development

**Figure 3:**
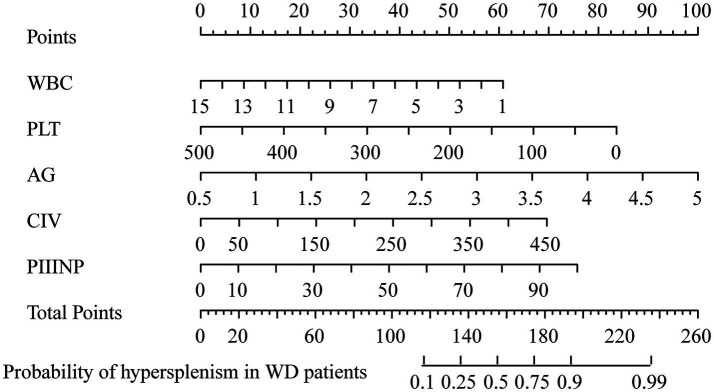
Line chart of the probability of splenomegaly occurrence in Wilson disease patients.

Subsequently, the VIF was used to assess multicollinearity among variables in the model. Results showed that all VIF values were less than 4, confirming the absence of multicollinearity among all variables in the model and indicating its robust statistical stability. We further evaluated the linear association between PIIINP and CIV using Pearson’s correlation coefficient. The results showed a weak positive correlation (r = 0.226, 95% CI: 0.126–0.321, *p* < 0.001), further confirming the absence of collinearity. To assess their individual and joint predictive value, we constructed univariate and bivariate logistic regression models on the test set. PIIINP alone achieved an AUC of 0.615 (95% CI, 0.527–0.704), while CIV alone yielded a higher AUC of 0.676 (95% CI, 0.592–0.760). The combined model showed a slightly improved AUC of 0.686 (95% CI, 0.602–0.771), indicating that PIIINP and CIV provide complementary rather than redundant predictive information. Finally, the predictive model was visualized using a nomogram, enabling quantitative prediction of the probability of splenomegaly occurrence in WD patients (Figure 3).

### 3.4 SHAP dependence analysis and feature interaction

**Figure 4:**
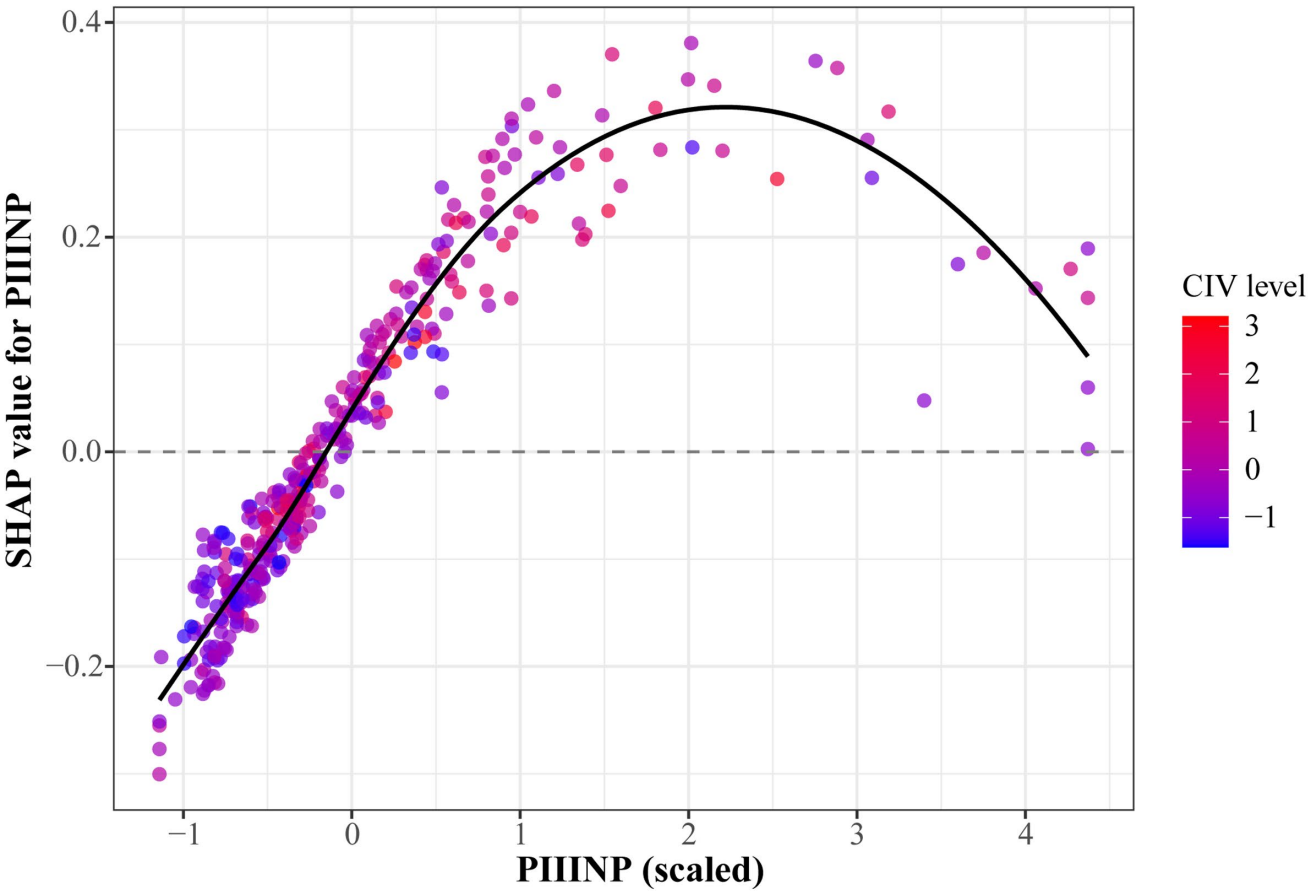
SHAP dependence plot for PIIINP colored by CIV.

To directly investigate whether the contribution of PIIINP is modulated by CIV—i.e., whether synergy or substitution exists—we performed SHAP dependence analysis for PIIINP using the fastshap package. Figure 4 plots the SHAP values of PIIINP against its own scaled values, with each point colored by the corresponding CIV level.

The SHAP value of PIIINP exhibited a non-linear pattern: negative contributions (SHAP < 0) were observed at scaled PIIINP values < 0, the strongest positive contributions occurred in the range of 0–2, and a decline was noted at values > 2. Importantly, across the entire range of PIIINP, points with different CIV levels were evenly scattered without any visible stratification or trend. This pattern indicates that the contribution of PIIINP is independent of CIV levels—i.e., no meaningful synergy or substitution exists between the two markers. The relationship is best characterized as additive rather than interactive.

### 3.5 ML model construction and performance comparison

To evaluate the discriminatory ability, calibration accuracy, and clinical utility of five ML models in predicting splenomegaly in WD patients, this study employed a validation approach using a “10-fold CV training set + independent test set.”

**Figure 5:**
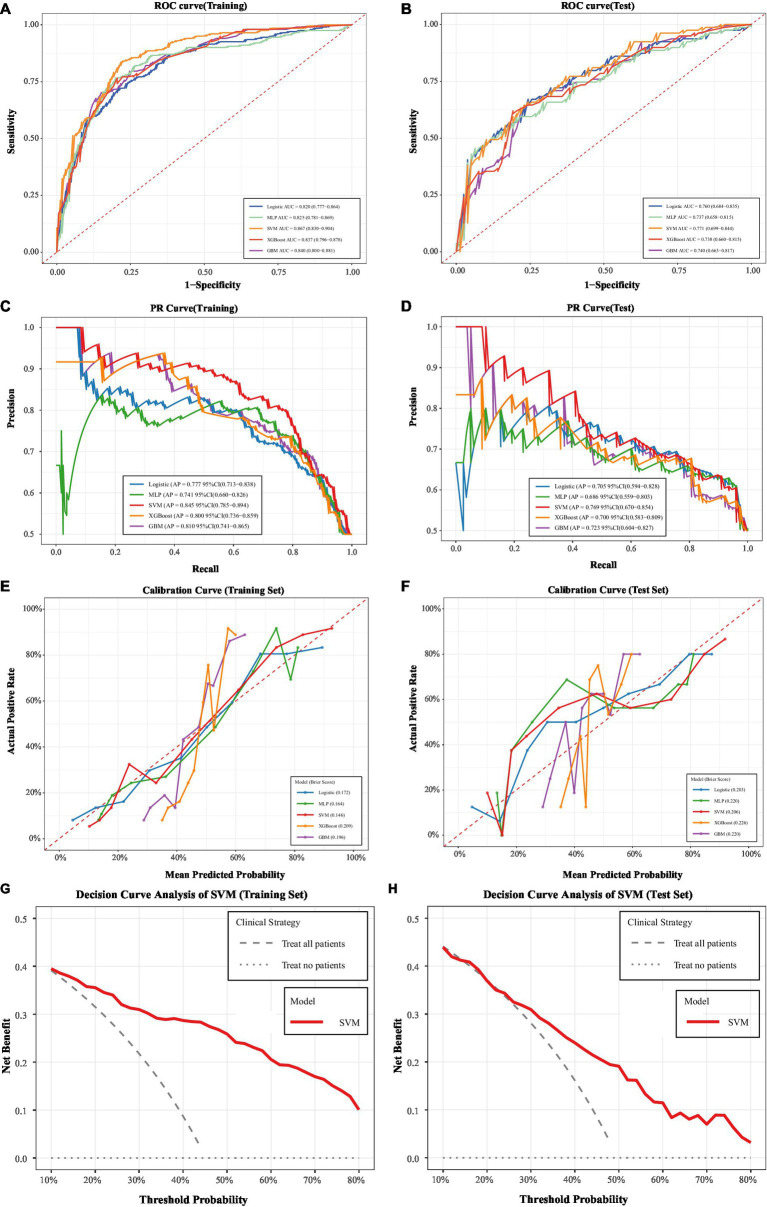
Comprehensive performance evaluation of machine learning (ML) models for hypersplenism prediction in Wilson disease patients. **(A)** ROC curve of multiple ML models (training set); **(B)** ROC curve of multiple ML models (test set); **(C)** PR curve of multiple ML models (training set); **(D)** PR curve of multiple ML models (test set); **(E)** calibration curve of multiple ML models (training set); **(F)** calibration curve of multiple ML models (test set); **(G)** decision curve analysis of the optimal SVM model (training set); **(H)** decision curve analysis of the optimal SVM model (test set).

Within the training set, the SVM model demonstrated optimal discriminatory ability, achieving an AUC of 0.867 (95% CI: 0.830–0.904), higher than other models. Concurrently, SVM exhibited outstanding overall classification performance, with accuracy (0.807, 95% CI: 0.775–0.847), specificity (0.856, 95% CI: 0.822–0.906), Precision (0.812, 95% CI: 0.755–0.872), and F1 score (0.777, 95% CI: 0.727–0.828) were the highest among all models, demonstrating excellent balance in positive and negative case classification (Figure 5A; Table 3). In the independent test set, although the discriminative ability of each model slightly decreased, overall generalization performance remained favorable: SVM still maintained optimal performance with an AUC of 0.771 (95% CI: 0.699–0.844) (Figure 5B; Table 3). ROC curve analysis of the test set further confirmed that models like SVM showed no significant degradation in discrimination ability, demonstrating robust generalization stability and consistent predictive value across independent WD patient samples.

**Table 3:** Performance metrics of candidate classification models for hypersplenism prediction in Wilson disease patients.

Classification model	AUC (95%CI)	Accuracy (95%CI)	Sensitivity (95%CI)	Specificity (95%CI)	Precision (95%CI)	F1 score (95%CI)
Training set						
Logistic regression	0.820 (0.777–0.864)	0.749 (0.714–0.786)	0.729 (0.666–0.776)	0.766 (0.713–0.821)	0.720 (0.662–0.800)	0.725 (0.673–0.767)
SVM	0.867 (0.830–0.904)	0.807 (0.775–0.847)	0.747 (0.682–0.818)	0.856 (0.822–0.906)	0.812 (0.755–0.872)	0.777 (0.727–0.828)
GBM	0.840 (0.800–0.881)	0.756 (0.706–0.794)	0.621 (0.515–0.698)	0.871 (0.820–0.919)	0.798 (0.714–0.863)	0.698 (0.613–0.764)
XGBoost	0.837 (0.796–0.878)	0.755 (0.717–0.787)	0.645 (0.558–0.715)	0.846 (0.802–0.880)	0.775 (0.696–0.843)	0.704 (0.635–0.760)
MLP	0.825 (0.781–0.869)	0.774 (0.745–0.814)	0.765 (0.700–0.833)	0.781 (0.735–0.827)	0.743 (0.679–0.807)	0.754 (0.707–0.801)
Test set						
Logistic regression	0.760 (0.684–0.835)	0.682 (0.613–0.745)	0.615 (0.525–0.682)	0.772 (0.656–0.844)	0.712 (0.621–0.837)	0.653 (0.581–0.737)
SVM	0.771 (0.699–0.844)	0.694 (0.637–0.760)	0.615 (0.515–0.700)	0.772 (0.659–0.862)	0.727 (0.623–0.864)	0.667 (0.576–0.766)
GBM	0.740 (0.663–0.817)	0.650 (0.575–0.706)	0.462 (0.389–0.546)	0.835 (0.740–0.914)	0.735 (0.602–0.872)	0.567 (0.486–0.669)
XGBoost	0.738 (0.660–0.815)	0.650 (0.586–0.701)	0.487 (0.391–0.571)	0.810 (0.703–0.888)	0.717 (0.586–0.846)	0.580 (0.475–0.659)
MLP	0.737 (0.658–0.815)	0.650 (0.578–0.725)	0.615 (0.514–0.699)	0.684 (0.572–0.785)	0.658 (0.572–0.778)	0.636 (0.545–0.720)

Considering the potential imbalance between positive and negative cases in clinical samples, the Precision-Recall (PR) Curve and Average Precision (AP) were further employed to evaluate the predictive quality of the models for patients with splenomegaly due to WD (Figures 5C,D). Within the training set, the AP values of all models ranged from 0.741 to 0.845, with SVM demonstrating optimal performance (AP = 0.845, 95% CI: 0.785–0.894), indicating the best balance between identification accuracy and recall for positive cases. In the test set, the AP values of all models ranged from 0.686 to 0.769, with SVM maintaining its lead (AP = 0.769, 95% CI: 0.670–0.854). The AP decay from the training to test set was controlled within a reasonable range, further validating the model’s reliable generalization capability.

Calibration curve analysis was used to evaluate the match between model-predicted probabilities and actual positive event occurrence rates (Figures 5E,F). The Brier score quantifies calibration error, with lower values indicating better calibration performance. Within the training set, SVM demonstrated optimal calibration (Brier score = 0.146), with its curve most closely approximating the ideal calibration line, indicating the highest alignment between predicted risk probabilities and actual event frequencies. In the test set, the Brier scores of all models increased slightly, consistent with normal fluctuations during generalization. However, SVM maintained excellent calibration performance (Brier score = 0.206) with no significant deviation in its curve. This confirms the reliability of its predicted probabilities in real-world applications, providing precise reference for clinical risk stratification.

Based on the above results, SVM was ultimately selected as the optimal predictive model for WD splenomegaly. To evaluate its practical value in guiding clinical intervention decisions, decision curve analysis (DCA) was further conducted. The training set DCA results (Figure 5G) show that within the threshold probability range of 10–60%, the net benefit curve of SVM exhibits a stable trajectory and remains significantly higher than the two extreme strategies — “treat everyone” and “treat no one”—throughout the entire range, demonstrating strong potential for clinical guidance. The independent test set DCA results (Figure 5H) further validated the model’s generalized practical value: although the SVM net benefit curve exhibited slight fluctuations in the low threshold range of 10–20%, its net benefit level remained consistent with the training set and showed stable trends within the core clinical decision-making range of 20–50%. The high alignment between the SVM net benefit curves in the training and test sets within the core intervention range confirms that SVM not only possesses strong clinical guidance value in the training set but also performs reliably in independent samples. This makes it a dependable reference for clinical intervention decisions in patients with splenomegaly due to WD.

### 3.6 Model interpretability analysis

**Figure 6:**
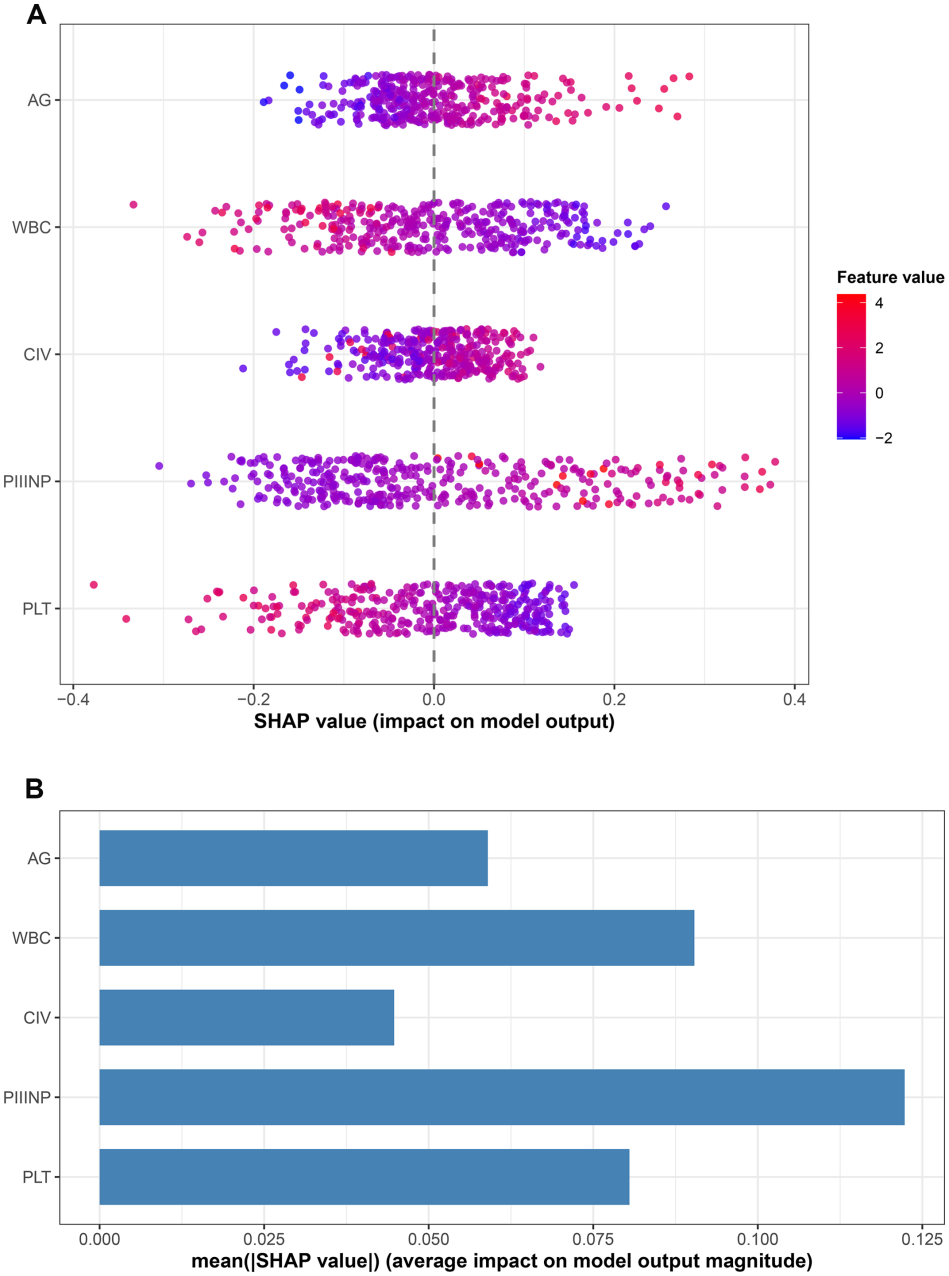
SHAP interpretability analysis of feature contributions for the SVM model in predicting splenomegaly in Wilson disease. **(A)** SHAP summary scatter plot, where each point represents a patient; the horizontal position reflects the direction and magnitude of the SHAP value’s impact on predicted risk, while the color gradient indicates the corresponding feature value; **(B)** SHAP feature-importance plot ranking variables according to their mean absolute SHAP values.

Additionally, the SHAP method was employed to analyze the feature contribution mechanism of the SVM classifier. A summary plot of SHAP value distributions (Figure 6A) was generated to visualize the impact of WBC, PLT, A/G, CIV, and PIIINP on predicting splenomegaly in WD. A positive SHAP value indicates that a higher value of that feature increases the risk of splenomegaly in WD patients, while a negative SHAP value suggests that a higher value of that feature reduces disease risk. Feature values are represented by color gradients, allowing further observation of how different feature levels modulate prediction risk. To quantify the overall contribution of each feature to the model’s predictive performance, this study calculated the average absolute SHAP value for each feature and plotted a feature importance bar chart (Figure 6B). Results showed that among the five included features, PIIINP had the highest average absolute SHAP value, followed by WBC, PLT, and A/G, while CIV had the lowest average absolute SHAP value. This finding confirms that PIIINP is the core feature for predicting splenomegaly in WD patients using the SVM model, providing a key reference for clinically identifying high-risk patients for splenomegaly in WD.

## 4 Discussion

WD, an autosomal recessive disorder caused by copper metabolism abnormalities, exhibits significant clinical heterogeneity and a dispersed onset pattern. Liver cirrhosis and splenomegaly associated with secondary portal hypertension represent core complications affecting patient prognosis, with their incidence increasing annually alongside liver fibrosis progression, substantially elevating risks of adverse outcomes such as bleeding and infection. Current clinical tools for liver function assessment, such as the Child-Pugh score and MELD score, primarily target patients with general liver cirrhosis (14, 15). They fail to account for the pathological feature of WD—copper accumulation-mediated specific liver fibrosis—and thus have limited predictive efficacy for splenomegaly. Previous studies have employed real-time two-dimensional shear wave elastography to assess liver stiffness in WD patients for predicting splenomegaly, achieving an AUC of 0.793 and demonstrating the predictive value of imaging metrics. However, this method relies on specialized ultrasound equipment and operator expertise, limiting its accessibility in primary healthcare settings (6). The training set AUC of the SVM model constructed in this study reached 0.867 (95% CI: 0.830–0.904), with a test set AUC of 0.771 (95% CI: 0.699–0.844). Although the test set AUC showed slight decline due to sample heterogeneity, it remained within the “good predictive performance” range of 0.7–0.9 (16). Moreover, the core advantage of this research model lies in its reliance on routine clinical tests such as complete blood count (WBC, PLT), liver fibrosis markers (PIIINP, CIV), and liver function indicators (A/G ratio). It requires no specialized imaging equipment, resulting in low testing costs and straightforward operation. This approach aligns well with the diagnostic realities of primary healthcare facilities, particularly addressing the clinical demands of rare diseases like WD, which exhibit “scattered incidence and necessitate widespread screening.”

In terms of ML algorithm selection, this study compared five algorithms—logistic regression, MLP, XGBoost, GBM, and SVM—and confirmed that the SVM model demonstrated optimal performance. This result aligns closely with the disease characteristics and data features of WD. As a rare disease, accumulating clinical samples for WD poses significant challenges. While the 524 samples in this study represent a relatively large cohort, they remain limited compared to common diseases. Furthermore, WD patients exhibit strong clinical phenotype heterogeneity, such as varying degrees of liver fibrosis and differing durations of copper chelation therapy. This results in data characterized by “small sample size, high dimensionality, and nonlinearity.” The SVM algorithm transforms high-dimensional nonlinear data into a low-dimensional linearly separable space through kernel function mapping. It also possesses regularization properties that effectively suppress overfitting (17). Its robustness in modeling small-sample, heterogeneous data has been validated in predictive model studies across fields such as hepatology and oncology (18, 19). In contrast, ensemble algorithms such as XGBoost and GBM may be prone to overfitting training-specific features in small-sample scenarios, leading to reduced generalization capabilities. Meanwhile, logistic regression, constrained by its linear assumption, struggles to capture the complex relationships of “multifactorial synergistic interactions” in the pathological progression of WD, resulting in slightly inferior predictive performance.

From the pathophysiological perspective of WD, the five core features identified in this study closely align with the disease chain of “copper accumulation - liver fibrosis - portal hypertension - hypersplenism,” further validating the biological plausibility of the model. *ATP7B* gene mutations cause copper transport dysfunction, leading to abnormal copper accumulation within hepatocytes. This triggers oxidative stress that damages hepatocytes, continuously activates hepatic stellate cells, and promotes the synthesis and deposition of extracellular matrix components such as type III and IV collagen, progressively advancing to liver fibrosis (20). Destruction of hepatic lobule architecture and capillaryization of hepatic sinusoids lead to a significant increase in portal venous resistance. Following the development of portal hypertension, impaired spleen venous return causes splenic congestion and splenomegaly. Widened splenic cords result in increased retention and destruction of blood cells within the spleen, ultimately manifesting as splenomegaly (21, 22). Among these, PIIINP, as a precursor peptide for type III collagen synthesis, has serum levels that directly reflect the degree of hepatic interstitial fibrosis. Interstitial fibrosis constitutes the core pathological change in WD cirrhosis, exhibiting a positive correlation with the severity of portal hypertension, which in turn drives splenic congestion and enlargement. (3, 23). Previous studies have confirmed that serum PIIINP levels in WD patients are significantly higher than in healthy individuals (24). In this study, PIIINP served as a core predictive feature, further validating the pathological logic of “liver fibrosis - portal hypertension - splenomegaly.” Elevated A/G ratio stems from reduced albumin synthesis due to hepatocyte damage in WD patients, coupled with compensatory increase in globulin production triggered by chronic inflammation. Both factors jointly contribute to splenomegaly by influencing the progression of liver fibrosis and the severity of portal hypertension (25). CIV, as the primary component of type IV collagen, exhibits elevated levels indicating hepatic sinusoidal capillaryization and the disappearance of fenestrations in sinusoidal endothelial cells. This further increases portal venous resistance and exacerbates splenic venous congestion (26). Together with PIIINP, they reflect the progression of liver fibrosis from the dimensions of “interstitial fibrosis” and “vascular structural remodeling”. However, in the multivariable SVM model, PIIINP’s predictive signal is amplified through synergy with WBC, PLT, and A/G, whereas CIV’s contribution is attenuated due to substantial overlap with these variables—highlighting the advantage of multivariable machine learning in capturing nonlinear interactions. WBC and PLT serve as direct laboratory indicators of splenic hyperfunction. During splenomegaly, enhanced macrophage activity within the spleen increases phagocytic destruction of granulocytes and platelets, leading to reduced peripheral blood levels (27–29). In this study, both markers were confirmed as protective factors: higher levels indicate milder splenic dysfunction. This finding aligns closely with clinical understanding, further validating the consistency between model characteristics and clinical diagnostic logic.

The clinical value of this study manifests in three key aspects: First, the constructed SVM model, based on routine diagnostic indicators, is simple to operate and cost-effective. It enables rapid risk stratification for splenomegaly in WD patients. High-risk patients can receive early interventions such as splenic artery embolization and enhanced copper chelation therapy, thereby reducing adverse outcomes like bleeding and infection. Second, the SHAP method addresses the “black box” challenge of ML models, identifying PIIINP as the core predictive feature. This provides a basis for targeted clinical monitoring of liver fibrosis indicators. Third, the model demonstrates robust generalization stability, laying a foundation for future widespread application. However, this study also has limitations. First, as a single-center retrospective study lacking multicenter prospective external validation, the model’s generalizability may be constrained. Second, dynamic factors such as treatment regimens and lifestyle were not incorporated. Future research should conduct multicenter, prospective studies incorporating WD patients from diverse regions and age groups to further validate the model’s generalizability. To better adapt to primary care settings, we will further explore a simplified scoring model based on categorical variables in subsequent research and expand the sample size for multicenter validation to facilitate the clinical translation of this simplified tool. Additionally, incorporating dynamic and genetic factors such as treatment regimens, lifestyle habits, and genetic polymorphisms to optimize feature sets could enhance predictive accuracy.

## 5 Conclusion

In summary, this study addresses the clinical challenge of “difficulties in early identification and lack of precise predictive tools” faced by patients with WD and splenomegaly. Using ML methods, we constructed and validated a predictive model for splenomegaly in WD patients. The SVM model was ultimately identified as the optimal solution. Analysis of calibration curves and DCA confirmed its excellent discriminatory ability, calibration accuracy, and clinical utility. Five key features—WBC, PLT, A/G, CIV, and PIIINP—selected via LASSO regression were identified through SHAP analysis, with PIIINP emerging as the core predictive indicator. This provides an objective quantitative basis for risk stratification of splenomegaly in WD patients. The findings offer a scientific tool for early identification, risk stratification, and precise intervention in splenomegaly among clinical WD patients, holding significant implications for improving patient prognosis.

## Data Availability

The original contributions presented in the study are included in the article/Supplementary material, further inquiries can be directed to the corresponding author.
