# Supplementary material for: Development and validation of a machine learning model for predicting hypersplenism in Wilson disease patients
Source: Front Med (Lausanne). 2026 Mar 18;13:1768024. doi: 10.3389/fmed.2026.1768024 (PMC13038950; doi:10.3389/fmed.2026.1768024)
Supplement: Supplementary file 1 [file Table_1.docx]

Supplementary Material

# Supplementary Notes on Five Machine Learning Methods

1. **Logistic Regression**

Logistic regression is a classical statistical model widely used for binary classification tasks, which is suitable for predicting the probability of a binary outcome (e.g., presence or absence of hypersplenism) in medical research. Unlike linear regression that outputs continuous values, it maps linear combinations of features to a probability between 0 and 1 using the sigmoid (logistic) function. The core formula of the sigmoid function is: $P(y=1|x) =\frac{1}{1+e^{-z}}$ Where $P(y=1|x)$ represents the probability of the positive outcome (hypersplenism) given input features $x$, and $Z$ is a linear combination of features $x$ and model coefficients $\theta$: $z = \theta_{0} + \theta_{1}x_{1} + \theta_{2} x_{2} + .... + \theta_{n}x_{n}$

During training, the model optimizes coefficients $\theta$ by minimizing cross-entropy loss, ensuring predicted probabilities align with actual outcomes. It is simple, interpretable, and computationally efficient, but assumes linear relationships between features and outcomes, which limits its performance in capturing complex nonlinear patterns in heterogeneous diseases like WD.

1. **Multilayer Perceptron (MLP)**

Multilayer Perceptron (MLP) is a type of feedforward artificial neural network (ANN) suitable for supervised learning tasks, including classification and regression. It consists of three hierarchical layers: an input layer (receives clinical features), one or more hidden layers (learns nonlinear feature representations), and an output layer (outputs classification results for hypersplenism).

The core mechanism relies on the backpropagation algorithm, which includes two key steps: **Forward propagation**: Input features are processed through hidden layers via activation functions (e.g., ReLU) to generate predicted outcomes. **Backward propagation**: The error between predicted and actual outcomes (measured by cross-entropy loss) is propagated backward to update weights and biases, minimizing cumulative loss.

MLP excels at learning complex nonlinear relationships and feature interactions, making it adaptable to high-dimensional clinical data. However, it requires large training datasets to avoid overfitting, and its "black-box" nature reduces interpretability—limitations that are particularly notable in rare disease research with limited samples like WD.

1. **Support Vector Machine (SVM)**

Support Vector Machine (SVM) is a powerful binary classification algorithm that performs well in small-sample, high-dimensional, and nonlinear data scenarios, which is well-suited for WD-related hypersplenism prediction (small sample size and heterogeneous clinical features). Its core goal is to find an optimal hyperplane that maximizes the margin between two classes (HG and non-HG groups).

For nonlinear data (e.g., complex correlations between liver fibrosis markers and hypersplenism), SVM uses kernel functions (e.g., radial basis function, RBF) to map input features into a high-dimensional linearly separable space. The model also incorporates regularization parameters (C) to balance classification accuracy and margin width, effectively suppressing overfitting.

During training, SVM minimizes the structural risk (instead of empirical risk) to enhance generalization ability. It has advantages of strong robustness to noise and high-dimensional data, but its computational complexity increases with sample size, and kernel parameter tuning requires careful cross-validation—consistent with the 10-fold cross-validation used in this study to optimize parameters.

1. **Extreme Gradient Boosting (XGBoost)**

Extreme Gradient Boosting (XGBoost) is an optimized ensemble learning algorithm based on gradient-boosted decision trees (GBDT). It constructs a strong classifier by sequentially integrating multiple weak decision tree classifiers, where each new tree corrects the prediction errors of the previous ensemble.

Key features of XGBoost include: **Gradient descent optimization**: Minimizes loss function (e.g., logistic loss for classification) by fitting the negative gradient of the previous error. **Regularization**: Adds L1 (Lasso) and L2 (Ridge) regularization terms to tree weights to prevent overfitting. **Handling missing values**: Automatically learns the optimal direction for missing feature values during training, adapting to incomplete clinical data.

XGBoost performs well in structured clinical data analysis, but in small-sample scenarios (e.g., WD research), it may overfit to specific features of the training set, leading to reduced generalization—consistent with the study’s finding that XGBoost’s performance was inferior to SVM in the test set.

1. **Gradient Boosting Machine (GBM)**

Gradient Boosting Machine (GBM) is a foundational ensemble learning algorithm that builds strong classifiers through iterative training of weak decision trees[11]. Similar to XGBoost, it uses gradient descent to minimize prediction errors, but lacks XGBoost’s optimized regularization and missing value handling mechanisms.

The training process of GBM involves: Initializing a weak classifier (e.g., a single-node decision tree) based on average outcome values. Iteratively training new trees to fit the gradient of the loss function relative to the current ensemble’s predictions. Weighting each tree by a learning rate and adding it to the ensemble to reduce cumulative error.

GBM can capture complex nonlinear relationships and feature interactions in clinical data, but it is more prone to overfitting than XGBoost in small-sample datasets. Its training speed is slower due to the lack of parallel computing optimization, which limits its efficiency in large-scale feature tuning.
